# Progress in Surgery

**Published:** 1899-04-15

**Authors:** 


### Progress in Surgery.

### GENERAL SURGERY.

(*Continued from page* 27.)

**Fractures and Dislocations—Lower Extremity.**—A rare instance of dislocation of the hip on to the dorsum of the ilium in a healthy lad, aged 13, is related by Mr. C. E. Bell.[5] The injury occurred at football and was treated as a sprain. At the end of five months, as the leg was of no use to him, being much shortened and absolutely incapable of bearing any weight, Mr. Bell made a free anterior incision, subperiosteally separated all the muscles attached above the lesser trochanter, and lifted the head out of its new bed. The articular cartilage was good, while the acetabulum was filled with new tissue and the remains of the capsular ligament. These having been removed with scissors and a scoop the cartilage seemed quite healthy. By steady traction and manipulation the head was replaced in the acetabulum. A long splint was applied for two months, the patient then being allowed up on crutches. Nine months after operation the boy had perfect movement of the limb and could stand and walk well. Dr. P. J. Timmins[16] describes a simple splint which he has employed for fractures of the femur in infants. After the child has been washed and rubbed with alcohol to harden the skin, a fairly stiff sheet of tin is cut from a paper pattern a little wider than the body to enable it to be bent up about an inch at the sides and extending from shoulders to the heels. After careful padding the chest and pelvis are fixed to the splint by two wide strips of adhesive bandage. The strip of splint which is to secure the broken leg is next flexed with the thigh and then bent back at the knee, leaving the femur portion long enough to serve for a fulcrum, on which may be extended into place the separated bone. Counter extension is secured by the already fixed pelvis. The femur and leg are strapped as needed, to the splint. The sound leg is done up in the same way, and then the body and both legs are bandaged as far as the feet. The child can now, secured as it were in a chair, be handled with ease by mother or nurse. Where angular deformity exists in ordinary fractures of the thigh, leg, or arm, or in ankylosed knee-joint, Dr. H. M. Hall[17] applies a plaster of Paris splint by a new method. While traction is made on the limb after reduction, a strip of cheese-cloth or common domestic cloth, one by two feet, is cut longways into a many-tailed bandage, and to the tails weights are attached. After the limb has been well padded the bandage with the weights is put astride it, and the plaster of Paris bandages applied from below upwards over the cloth in between its tails, which are thereby converted into rounded cords. The plaster having hardened, the tails with the weights are cut off close to it. The ends retract within the plaster, and leave small holes, which are useful for ventilation. In the treatment of fractured patella by open operation, I. S. Haynes[38] operates after a week has elapsed by means of the purse-string suture. The suture he employs is of strong braided silk, capable of standing a heavy strain. Two pieces, each eighteen inches long, freshly sterilised, are used, threaded into long, half-curved surgical needles. The upper fragment is first semi-encircled. The needle is entered in the midst of the tissues close to the fractured edge of the patella, carried upwards, with its point closely hugging the bone, and brought out at the lateral and superior angle; it is reinserted close to the hole of emergence, and carried through the tendon of the quadriceps extensor, close to the bone, then brought out at the other lateral and superior angle; then it is passed and made to emerge at the side of the fractured edge opposite to that of entrance. The lower fragment is similarly semi-encircled by the other piece of silk, which also keeps close to the bone transfixing the ligamentum patellæ. The two sutures are tied simultaneously with as much force as the silk will stand, and the fractured surfaces brought accurately together. He rightly considers that an important point is the removal of all the torn shreds of tissue between the fragments and the close suturing of the lacerated soft parts, and adds one precaution—namely, to handle the tissues as little as possible, and then with instruments in preference to the fingers, and most of all to do the handling yourself. Sir Wm. Stokes[19] thinks that this circumferential suture, whether carried out by the open or subcutaneous method, open to the objection of probably interfering by pressure with the nutrition of the bone. He makes a vertical incision in preference to making a flap, and drills the fragments for the introduction of the sutures with a drill worked by an electro-motor. He believes that the open method should be adopted in all cases of fractured patella, recent as well as old, unless some distinct contra-indication for operation exists. He has treated five cases by this method and suture with satisfactory results in all except one, in which suppuration occurred. Barker's method of suture by passing a wire behind both fragments did not commend itself to him. Dr. Martin[40] thinks that arthrotomy with bony suture is the only plan capable of re-establishing in its integrity the extensor apparatus of the lower limb. It should not be attempted after the fiftieth year, and only when there is a separation of three centimetres or more between the fragments, with marked effusion into the joint. The patient should be warned of the risks of operation and allowed to choose according to his profession, &c. Since Dr. C. Phelps[41] in 1890 related 42 cases and exhibited 22 in whom osseous union was demonstrable two months to five years after operation, the number operated upon by him has been increased to 105. Apparent osseous union occurred in all. He is convinced that in all cases in which osseous apposition is assured, sepsis avoided, and immobility maintained, osseous union will be effected, and he sustains the contention that, with proper aseptic precautions, operative treatment is entirely devoid of danger to life. He considers that the time has already arrived when the final verdict upon the method of suture may be safely rendered. Mr. W. H. Battle[42] records a case of compound fracture of the patella due to a kick from a horse, in a cabman aged 53. Antiseptics were immediately applied, and about five hours later the joint cavity, the bony fragments and surrounding parts were thoroughly cleansed with perchloride of mercury solution, the fragments sutured with silver wire, and a drainage tube inserted on the outer side of the joint. About a month later the patient was discharged with firm union of the patella and able to walk with a movable joint. Dr. R. W. Knox[43] believes that the old method of plaster of Paris dressing is the best splint for fractures about the leg and ankle, and that these should be put up as in the typical Pott's fracture immediately or directly after the receipt of the injury and reduction of the fragments. The tendency to displacement of the foot outwards and backwards is prevented by this means. Swelling is not a contra-indication to this treatment. Kummer[44] has collected from medical literature 90 cases of fracture of the astragalus. The following varieties occur: (1) Fracture of the neck (most frequent) and often accompanied by fracture of the malleoli, or os calcis; (2) horizontal; (3) sagittal; (4) oblique; (5) comminuted fracture; and (6) fracture of the posterior apophysis or os trigonum. As to treatment, immobilisation, with the foot flexed to a right angle, with massage and gentle movement after the first few days, should be adopted. In fracture of the neck of the bone, with irreducible displacement, the removal either of the head or of the body of the bone has given good results. In fracture of the apophysis, failing attempt to secure bony union, this process should be removed. An interesting case of fracture of the os calcis is reported by MM. Tuffier and Desfosses.[45] The skiagraph showed that a triangular portion of the bone, containing the attachment of the tendo-Achilles, was torn away, evidently by muscular violence, and also that the line of fracture corresponded to the lines of the structure of the bone, and that the break started at the point where the force was applied to the longitudinal fibres of the bone.

[35] Lancet, Oct. 22, 1898, p. 1059. [35] New York Med. Rec., Sept. 24, 1898, p. 455. [37] Ibid, p. 444. [38] New York Med. Jour., Sept. 3, 1898, p. 334. [39] Brit. Med. Jour., Dec. 3, 1898, p. 1668. [40] New York Med. Rec., Dec. 10, 1898; and Archiv. Med. Belges, June. [41] New York Med. Jour., Dec. 17, 1898, p. 871. [44] Lancet, Sept. 10, 1898, p. 690. [43] New York Med. Rec., Sept. 17, 1898, p. 403. [44] Med. Chron., Aug., 1898, p. 387. [45] Amer. Jour. Med. Sci., Aug., 1898, p. 230.

